# Leveraging the new with the old: providing a framework for the integration of historic microarray studies with next generation sequencing

**DOI:** 10.1186/1471-2105-15-S11-S3

**Published:** 2014-10-21

**Authors:** Michael A Bauer, Shweta S Chavan, Erich A Peterson, Christoph J Heuck, Donald J Johann

**Affiliations:** 1 Myeloma Institute for Research and Therapy, University of Arkansas for Medical Sciences, Little Rock, AR, USA

## Abstract

Next Generation Sequencing (NGS) methods are rapidly providing remarkable advances in our ability to study the molecular profiles of human cancers. However, the scientific discovery offered by NGS also includes challenges concerning the interpretation of large and non-trivial experimental results. This task is potentially further complicated when a multitude of molecular profiling modalities are available, with the goal of a more integrative and comprehensive analysis of the cancer biology.

Microarray transcriptome analyses have resulted in important advances in both the scientific and clinical domains of biomedicine. Importantly, as technology advances, it is critical to leverage what has been gained from historic approaches (e.g., microarrays) with new approaches (NGS). In this regard, necessity dictated a need to utilize and leverage the many years of historical microarray data with new NGS approaches. This is especially important since NGS approaches are now entering clinical medicine. For instance, NGS-based comprehensive analysis of certain cancers has already helped to uncover specific mutations that contribute to the malignant process, identify new therapeutic targets, and improve opportunities for choosing the best treatment for an individual patient.

A suite of custom software tools have been developed to rapidly integrate, explore, discover and validate molecular profiling data from the NGS modalities of Whole Exome Sequencing (WES) and RNA-seq with each other, as well as with historical microarray and salient clinical datasets. Importantly, our approach is independent of any particular type of NGS suite(s) or cancer types. This novel bioinformatic framework is now assisting with the scientific and clinical management of patients with multiple myeloma.

## Background

Next generation sequencing (NGS) is a new frontier in cancer and biomedical research, and these approaches are rapidly becoming the preferred method for human disease-based analysis due to vastly improved genome coverage and resolution [1]. Recently, the FDA granted marketing authorization for the first high-throughput NGS system (Illumina MySeqDx) for the purpose of clinical test development [2]. This FDA decision is clearly related to the fact that NGS methodologies are enabling a new understanding of cancer. For instance, recent work from The Cancer Genome Atlas (TCGA) has shown that a particular cancer tissue of origin may be less relevant to therapeutic response and prognosis than the collection of causative mutations [3]. Thus, for cancer scenarios where the standard-of-care options are poor (e.g., drug resistance, metastasis), drug assignments based on the mutational landscape of a patient's tumor can sometimes provide significant benefit, and are a very active research topic in clinical trials and translational medicine [4]. However, NGS also brings new demands regarding the size and complexity of the associated data sets. These "big data" challenges are further magnified when multiple NGS modalities are utilized and there are needs or requirements to integrate this data with other molecular profiling techniques (e.g., microarrays).

NGS and related studies have already contributed significantly to the improved understanding of multiple myeloma (MM) [5-7]. Most recently there were 203 paired tumor/normal DNA samples analysed by either Whole Exome Sequencing (WES) or Whole Genome Sequencing (WGS) [8]. A principal finding was a very complex genetic landscape with extensive clonal heterogeneity that serves to limit scientific and clinical utility. In this case it appears DNA analysis is necessary but not sufficient. An approach to the genetic complexity and heterogeneity issue is to combine additional modalities, for example transcriptome data. By enhancing a DNA examination with transcriptome data, a multi-modality study of a particular patient's cancer is formed, yielding increased scientific/analytical rigor and potential insights. This is a primary aim of our methodology.

Microarrays have contributed significantly towards an improved understanding of MM and many other cancers, and there are large archives in the private and public domains. Thus, a familiarity with the analytic nature of transcriptome data from different modalities is important, especially regarding the explanatory abilities for cancer biology questions. To this end, a comparison of microarray vs. RNA-seq is provided in additional file 1[9]. In contrast to RNA-seq, microarray data is compressed and is largely a correlative science. RNA-seq requires less sample material, has base level resolution, a much larger dynamic range, is discovery-based for both novel isoforms and gene fusions, and can distinguish known splice isoforms.

For a cancer center or institute that has a focus on a particular cancer type, and who have a large volume of microarray data, the evolutionary shift in technology (i.e., microarray to NGS) can have disruptive effects on scientific and clinical workflows. Hence, there is a significant need to integrate and leverage historic molecular profiling studies (e.g., microarray) with new and existing NGS modalities. Additionally, the ability to capture, model, and successfully carry forward key aspects of a particular cancer biology learned from the legacy data and incorporate this knowledge into new and evolving software approaches is challenging, but needs to be carefully considered.

This study is a continuation of efforts to develop efficient software methodologies to allow for the incorporation and analysis of experimental data from a variety of molecular profiling modalities [10]. The current and historical work involving microarray technology to study the cancer biology of MM at the Myeloma Institute for Research and Therapy (MIRT), at the University of Arkansas for Medical Sciences (UAMS), has resulted in over 19,000 microarray studies. This has included important insights into the cancer biology of MM, for example, the development of risk scores [11,12] and a molecular classification [13]. A significant need exists to leverage this important work learned and gained from microarrays with the new and evolving NGS modalities.

**Figure 1:**
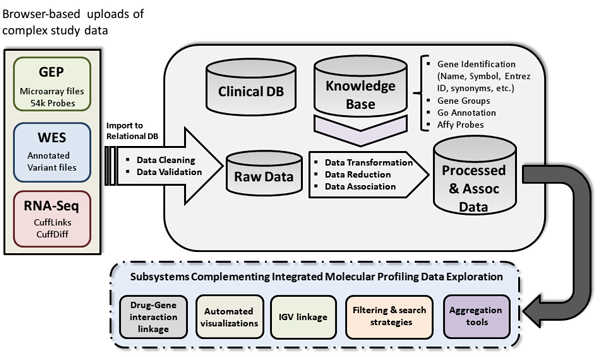
**NGS Association System Information Architecture**. The basis for integration of multiple molecular profiling modalities is illustrated. This includes processes for data transformation, reduction and association, as well as the direct interfacing to multiple custom and third party software tools and subsystems.

Using a three tier approach the software has been redesigned, and extended to accommodate a variety of integration scenarios involving molecular profiling experimental data from **i) **microarrays, **ii) **RNA-seq, and **iii) **Whole Exome Sequencing (WES), as well as features to capture and model significant cancer biology items (e.g., prognostic gene groups and molecular classification). Figure 1 illustrates the information architecture of our NGS Association System. Importantly, our approach is independent of any particular type of transcriptome reconstruction, variant caller or annotator, quantitation tool, or file format. Input requirements simply consist of a tab delimited file with column one being a Gene ID (Ensembl, Entrez, or Genbank Accession) if known, column two being the gene symbol (if known), and column three a chromosome plus loci pair (if known). Additional columns are imported and associated in a set-based manner. We continue to integrate Affymetric probe set-IDs, and gene annotation information from an assortment of sources as previously reported, as well as employ a variety of strategies to maximize the integration, annotation, and association of biological information [10].

The novel aspects of our approach are the abilities that allow for a ***rapid integration, exploration, discovery and validation ***of large and complex experimental data from a wide variety of NGS and microarray molecular profiling modalities. For instance, the approach is useful for comparing the expression of genes of interest between RNA-seq and microarray. Other supported comparisons are: **i) **Whole Exome Sequencing (WES) vs. Microarray, **ii) **WES vs. RNA-seq, **iii) **WES vs. RNA-seq vs. Microarray (triple integration). The triple integration approach is illustrated in the paper since it is the most difficult. To our knowledge, there is no existing software suite available, commercial or open source, which provides the triple integration feature. These abilities enable a more informed view of the intricate details present in the complex cancer biology, especially in cases of extensive heterogeneity. Finally, our approach is independent of any particular cancer type. Multiple myeloma is used only as an illustrative example. Eventually, we plan to make our software available to the greater scientific community, possibly as open source.

## Methods

### Sample descriptions and library preparation

Bone marrow aspirates and one peripheral blood sample were collected at UAMS, MIRT from a single patient diagnosed with MM and three normal donors. The sample collection protocol was approved by the UAMS Institutional Review Board (IRB). Plasma cells from bone marrow aspirate samples were enriched by anti-CD-138 immuno-magnetic bead selection in a central laboratory as previously described [14]. CD-138 is a marker for a malignant plasma cell, and for this sample the degree of CD-138 purification was 95%. Germline material was obtained from the buffy coat following density gradient centrifugation of the peripheral blood sample. To ensure the absence of plasma cells, buffy coat material was also examined by flow cytometry. Microarray data files were obtained from the study archive section in our MM historical database and were processed as previously reported [12]. Note, GEO-based extraction using Bioconductor with GEOquery along with in-house developed data wrangling tools can also be used in workflows, provided the output is in a tab or comma separated value format in the order of : *gene, probeID, value *[15].

Whole exome capture libraries were constructed from 500 ng of tumor and normal DNA after shearing, end repair, phosphorylation, and ligation to bar coded sequencing adaptors for a single patient. DNA was fragmented by the S220 focused-ultrasonicator (Covaris), using a standard protocol for a target bp of 300. DNA was size selected for lengths between ~250 - 330 bp and subjected to exonic hybrid capture using SureSelectXT Human All Exon V5 (Agilent). Libraries were enriched using 14 cycles of PCR. The library was run at a concentration of 8 pM. Samples were multiplexed and sequenced on an Illumina HiSeq 2500 using the rapid run mode (paired-end 101 bp reads) to an average depth of coverage of 100x, for tumor and normal respectively.

For the RNA-seq samples each Illumina mRNA-seq library was prepared using the TrueSeq mRNA kit v2. The starting sample material was 200 ng of total RNA and fashioned according to the manufactures instructions. This was done for one patient with MM and three normal donors. Poly-A selection for mRNA isolation using streptavidin-coated magnetic beads, followed by thermal mRNA fragmentation per standard Illumina protocol, was used during the sample prep process. The fragmented mRNA was subjected to cDNA synthesis using reverse transcriptase according to the manufacturer's instructions. The cDNA was then converted into double stranded cDNA followed by an end repair process, and then ligated to Illumina paired end (PE) adaptors. Size selection was performed using AMPure XP beads (Beckman Coulter), generating cDNA libraries ranging in size from 300 to 350 bp (base pairs). The library was enriched using 15 cycles of PCR and purified again by AMPure XP beads. The library was run at a concentration of 8 pM. The RNA-seq experiment was run on an Illumina HiSeq 2500 in the rapid run mode utilizing 101 bp PE sequencing. In summary, the fragment length is ~300-350 bp and thus the inner-mate-distance is ~98-148 bp since we are performing 101 bp PE sequencing.

### Whole exome sequencing (WES) data analysis and alignment

Generation of FASTQ files was performed via CASAVA (http://support.illumina.com/sequencing/sequencing_software/casava.html). Reads were analyzed and quality checked using FastQC (http://www.bioinformatics.babraham.ac.uk/projects/fastqc/). Based on the quality reports, it was decided to trim the last nine bases from each read, using Trimmomatic [16]. Paired-end reads were aligned to the human genome (GRCh37) by a hybrid approach that utilizes BWA [17] and Stampy [18]. Duplicate reads were removed using Picard tools (http://picard.sourceforge.net). Sequence recalibration and local realignment were performed using GATK [19]. Single nucleotide variant (SNV) and small insertions and deletions (InDels) calling was performed by Strelka [20]. SnpEff [21] was used to functionally annotate all variants.

### RNA-seq read alignment and transcriptome processing

The RNA-seq data analysis utilized the Tuxedo suite. A standard pipeline protocol was utilized. Trimmomatic was utilized for preprocessing prior to alignment. RNA-seq reads were mapped using TopHat version 2.0.8 (http://ccb.jhu.edu/software/tophat/index.html) against the human genome (GRCh37). Alignment utilized the following options: "-p 8 -G Ensembl.gtf", where Ensembl.gtf contains the coding transcripts in GTF format. Cufflinks version 2.1.1 (http://cufflinks.cbcb.umd.edu/) was run for transcriptome reconstruction and quantitation for each generated BAM file with the following options: "-p 8 -g Ensembl.gtf", thus allowing for both annotation and novel transcript discovery. Normal donor assemblies were merged with Cuffmerge. Differential expression analysis between the MM sample and normal donor pool utilized Cuffdiff.

### System session management, data import, processing, and user interface

#### User profile management

Additional file 2 illustrates the user profile management feature of our system. **Subfigure A **shows the User Profile tab. In this case, the "Login Name" is utilizing the UAMS domain account, which provides extra security and ease of use for this particular user. Various privileges for site administrator rights, as well as allowing for special debug modes, and IGV (Integrative Genomics Viewer) [22] integration are provided. **Subfigure B **displays the Gene groups tab that allows for the creation and modification of custom groups of genes for user driven search strategies.

#### Upload of experimental data

Additional file 3 illustrates the experimental upload features. Provisions are provided to create new patient (or a symbol for a scientific study, e.g., mouse model, cell line, etc.) entries and this is shown in **subfigure A**. One or more experiments along with salient information can then be associated with a patient, etc. This is shown in **subfigure B**. Due to the large size of datasets, we have developed a scheduling strategy to accommodate data import and associative processing, which can be time intensive. Depending on the particular molecular profiling modality, one or several files may be uploaded to a server. Next, an *import *request is placed in a job queue. The import process will begin some of the basic data validation steps pertaining to the molecular profiling experimental data, and place it into a staging/raw set of database tables. Subsequently, the newly imported experimental data can now be subjected to a variety of transformations, associations, reductions, filtering operations, etc. The import task/process continually checks the queue, and processes the next available job, by either importing or transforming the user requested datasets. This is illustrated in **subfigure C**.

#### Data cleaning and validation

Data integrity is paramount for accurate and reproducible science. In biomedicine and life sciences research, this can be a particularly challenging endeavour, given the arrival of *big data *from Next Generation Sequencing (NGS) experiments. The data is so massive that is becomes easy for errors to hide from detection. Thus, all files produced by the different NGS modalities were studied, and a specific parser has been designed for that file type. Some of the basic cleaning and validation steps include: giving the generic text data an explicit data type, range checks on all numbers, set membership checks on various symbol names/groups, etc. Incoming data must fit within certain expected ranges and formats before being placed in the appropriate database table. Aspects of these operations are shown in Figure 1.

#### Data transformation, reduction, and management

Once the data has successfully been imported into the database in its raw format, the next step is to perform the association and transformations to annotate and connect the different datasets. The sources of information used to construct our knowledge base, as well as the operation of the association algorithms, are modifications and enhancements of previously described methods [10]. Newly created ID association tables and software, acting as a switching center, allows for the conversion between different gene identifiers, and is shown in additional file 4. Conversion can be either direct or indirect depending on starting and target IDs. The ability to also convert between the different identifiers based on chromosome and loci has been added.

An executive console is illustrated in additional file 5. This feature provides the ability to view experimental/study information on an aggregate basis, along with the particular modality, date loaded, number of rows (size) of various tables, etc. The ability to perform complex cascading delete operations on an entire study or just certain aspects is provided.

#### User interface

Different experiments and modalities can be associated with a particular patient. On the patient view, available experiments may be viewed individually or in different combinations with other existing modalities, giving an integrated view. Additional file 6 displays an example of the Patient Experiments View, where for a particular patient, the available studies (RNA-seq, DNA, Microarray) for integration and viewing may be chosen. The user simply checks one or more of the left most boxes (next to Date), and then presses a "View" button. Additional file 7 contains a figure that shows results from the Cuffdiff Experiment for patient John Doe (Date 3/26/2014) following a simple selection and view. **Subfigure A **shows the initially loaded dataset and **subfigure B **shows a reduced dataset after applying a user defined custom filter consisting of five key genes.

Since users of the system will have different backgrounds and needs (e.g., clinical researcher vs. basic scientist), the ability to show a minimum or maximum number of columns/data elements has been provided. This is illustrated in the figures contained in additional files 8 and 9. Basic ergonomics are utilized regarding the display of grid columns, specifically column color schemes and their grid location, and are consistent regardless of the study, or studies loaded. For instance, the first three columns and last column are always: *Entrez ID, Gene Symbol, Gene Name*, and *Member List*, respectively. These are always colored grey in the referenced figures. *Member List *indicates the gene belongs to a certain user defined group(s), it is color coded but also has a "hover over" feature to reveal the group name (since gene groups may be shared among users). WES data/columns will appear next (if loaded) and these columns are always shaded green. RNA-seq columns then follow (not loaded in this example) and would be shaded light red. Finally, microarray columns appear last and are always shaded blue.

#### Data visualization

In addition to seeing the data in tabular formats, dynamically produced visualizations are created for different modalities to aid data explorations. Currently, these visualizations include simple bar and pie charts. These data-driven visualizations charts are created using NVD3 (http://nvd3.org) and D3 (http://d3js.org), and are demonstrated later in this study.

#### IGV integration

An integration option with IGV to promote the exploration and validation of the NGS data has been further developed. Using the IGV port interface the server sets up a socket connection to the users running a copy of IGV. Through this connection the server sends commands to IGV to load datasets for a particular NGS experiment. This automation allows the user to quickly jump to different locations within the genome, by a single click on the gene(s) of interest, in the web browser. Illustrated examples of this feature will appear in the results section.

#### Systems integration testing

A systems integration testing strategy was developed owing to the complexity of the NGS Association System software. Due to the utilization of third party tools and their custom developed interfaces, different software technologies (e.g., database stored procedures for fetching and set-based operations on large data sets, object oriented approaches for business logic, and a modern browser-based presentation system); along with extensive extraction, translate, and load (ETL) operations for the various molecular profiling modalities, as well as knowledge base construction and association operations, unit testing alone was not sufficient. Thus, a series of more elaborate testing scenarios are employed to exercise the entire NGS Association System in very deliberate ways, along with expected system actions and outputs.

## Results and discussion

In this study, custom software methodologies have been further developed to allow for the rapid integration, exploration, discovery and validation of experimental data from a variety of molecular profiling modalities [10]. The current and historical work involving microarray technology to study the cancer biology of MM at MIRT has resulted in an extremely large microarray archive. Certainly, there is a need to leverage this important work with the new and evolving NGS modalities. Methods to incorporate cancer biology knowledge garnered from historic microarray studies, and to best aid with analysis approaches involving new NGS modalities, continue to be an active research topic. Importantly, for cancers known to be very genetically complex and heterogeneous (e.g., MM), DNA/WES is necessary but not sufficient, and an integrated multi-modality approach as presented in this study may aid with this dilemma.

**Figure 2:**
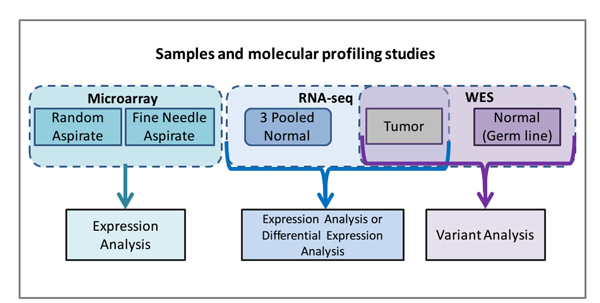
**Samples and Molecular profiling studies**. Illustrated is a diagram of the patient samples and molecular profiling studies used in the various analyses.

The NGS Association system is independent of any particular cancer type. Molecular profiling results from a single patient with MM have been selected to illustrate our systems ability to rapidly find and analyse salient findings across multiple modalities. A diagram of the samples and molecular profiling studies used in the various analyses are presented in Figure 2. Although the outcomes for MM patients have considerably improved, those who are refractory to, or relapse following therapy with an IMiD and/or proteasome inhibitor have a poor prognosis [23]. NGS sequencing of MM patients have revealed multiple targetable mutations, including KRAS [5,8].

**Figure 3:**
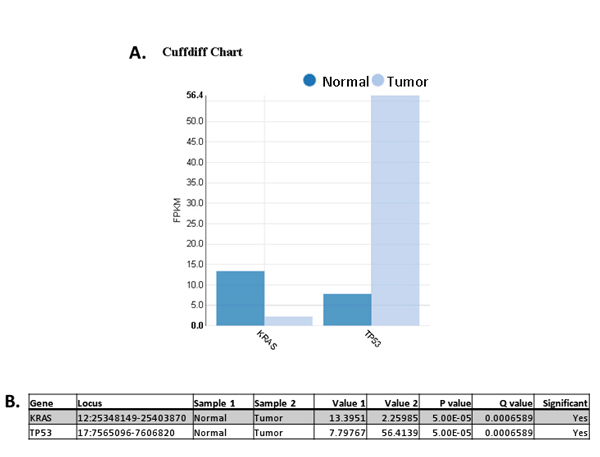
**System Rendered Results for Key Cancer Biology Genes**. Automatically rendered bar chart and table from a Cuffdiff dataset that was searched using two genes (*KRAS *and *TP53*), which have cancer biology significance in many cancers, including MM. The Cuffdiff Chart in **subfigure A **was quickly generated by a single point and click. All genes in the table (**subfigure B**) contain a significant p-value and q-value.

**Figure 4:**
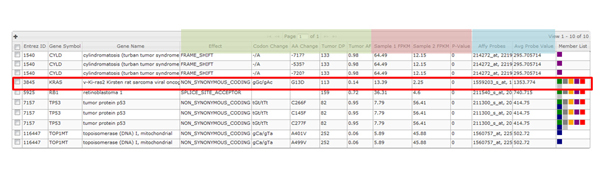
**Triple Integration (WES, Cuffdiff Gene, Microarray)**. A triple integration was performed by combining Cuffdiff gene data with WES and a gene expression microarray experiment. The integrated WES data (green columns) includes: *Effect, Codon Change, AA Change, Tumor DP *(Depth), *Tumor AF *(Allelic Frequency). The corresponding RNA-seq data (light red columns), show the *Sample 1 FPKM *(pooled normal), *Sample 2 FPKM *(tumor), and *p-value *from Cufflinks. The microarray data (blue columns) display the corresponding *Affy Probes*, and *Avg Probe Values*. The grey columns are common across all modalities and show, *Entrez ID, Gene Symbol, Gene Name*, and *Member List *(gene list).

Figure 3 shows the system rendered results of the Cuffdiff dataset that was searched using two genes having cancer biology significance in many cancers including MM (*KRAS *and *TP53*). The Cuffdiff Chart in **subfigure A **was automatically generated by a single point and click. All genes in the table (**subfigure B**) contain a significant p-value and q-value. Next, a triple integration was performed by combining the Cuffdiff gene data with WES and a microarray experiment. This is shown in Figure 4. Here the search criteria was based on a user defined five key gene group, which includes *KRAS *and *TP53*. The *KRAS *entry is circled for illustrative purposes.

The integrated WES data (green columns) includes: *Effect, Codon Change, AA Change, Tumor DP *(Depth), *Tumor AF *(Allelic Frequency). Next, the corresponding RNA-seq data (light red columns) show the *Sample 1 FPKM *(pooled normal), *Sample 2 FPKM *(tumor), and *p-value *from Cufflinks. The microarray data (blue columns) display the corresponding *Affy Probes*, and *Avg Probe Values*. This minimal view shows *KRAS *with a non-synonymous hotspot mutation resulting in a change in the amino acid Glycine to Asparatic Acid at the 13^th ^position (G13D). This finding has a depth of coverage of 113 reads and an allelic frequency of 14%. This is a known druggable mutation. The table in additional file 10 shows potential drug candidates through drug-gene interactions via custom automation/linkage (Figure 1, Subsystems Complementing Integrated Molecular Profiling Data Exploration, Drug-Gene interaction linkage) from our system to the DGIdb [24].

**Figure 5:**
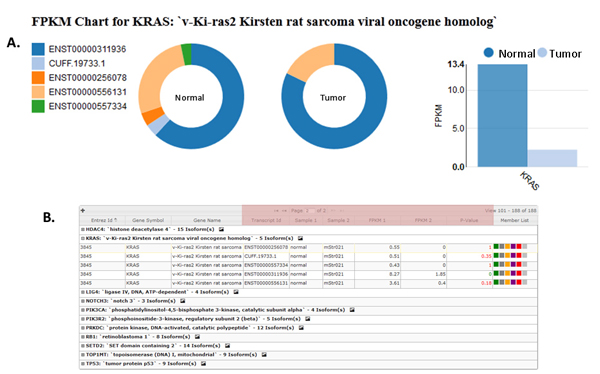
***KRAS *Automated Visualization and Exploration**. **Subfigure A **shows an automated visualization of the FPKM Chart for *KRAS *and its associated isoforms. This was automatically generated by clicking the picture icon located in the rightmost region on the first line of the KRAS entry, in **subfigure B**. In this view, the novel isoform (i.e., CUFF.19733.1) was also fetched from the experimental data. The columns *Transcript Id *and *P-Value *show that only one transcript (ENST00000311936) is significant.

Additional file 11 shows a triple integration of data sets from WES, Cuffdiff isoforms, and gene expression microarray. There are two *KRAS *gene isoform transcripts from the Ensembl annotation with non-zero values in the column *Sample 2 FPKM *(tumor), namely ENST00000556131 and ENST00000311936. Figure 5, **subfigure A **shows an automated visualization of the FPKM Chart for *KRAS *and its associated isoforms. This was automatically generated by clicking the picture icon located in the rightmost region on the first line of the *KRAS *entry, in **subfigure B**. In this view the novel isoform (i.e., CUFF.19733.1) was also fetched from the experimental data. The columns *Transcript Id *and *P-Value *show that only one transcript (ENST00000311936) is significant. The visualization allows changes to be spotted quickly. A "hover over" feature, allows viewing the tumor or normal isoform FPKM values, in the ring/donut plot of **subfigure A**.

**Figure 6:**
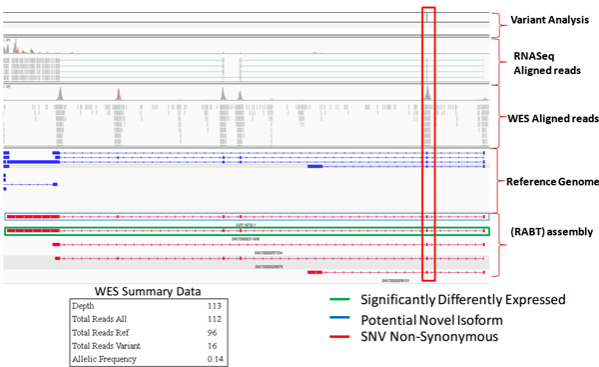
**Validation of the *KRAS *G13D Hotspot Mutation via IGV Automation**. IGV automation is utilized to validate the non-synonymous hotspot mutation involving codon 13 for *KRAS*, where Glycine is replaced by Aspartic Acid (G13D). By a single point and click on the web page entry for *KRAS*, the IGV display is rendered. During this process, all relevant information is spontaneously sent to IGV. This includes data from Variant Analysis, RNA-seq aligned reads, WES aligned reads, the reference genome and RABT assembly. For illustrative purposes, the significant isoform, ENST00000311936, is circled in green, and the location of the G13D hotspot mutation is circled in red. A potential novel isoform (CUFF.19733.1) from the normal pool is circled in blue.

Further discovery and validation of the *KRAS *finding is illustrated in Figure 6. IGV automation is utilized, and by a single point and click on the web page entry for *KRAS*, relevant information is spontaneously sent to IGV. The transferred information includes data from Variant Analysis, RNA-seq aligned reads, WES aligned reads, the reference genome and RABT (Referenced Annotation Based Transcript) assembly. For illustrative purposes, the significant isoform, ENST00000311936, is circled in green, and the location of the non-synonymous hotspot mutation, G13D, is circled in red. A potential novel isoform (CUFF.19733.1) from the normal pool is circled in blue.

Further exploration, discovery, and validation is accomplished by using IGV to drive further into the integrated complex data sets, as is shown in additional file 12. The same circled entries and color assignments apply. IGV is used to validate the *KRAS *G13D hotspot mutation by examining the WES aligned reads. Since *KRAS *is known to code from the reverse strand, the point mutation is shown as a "T" on the IGV display. However, the RNA-seq aligned reads do not appear to show evidence of the point mutation. This is confirmed by driving further into the IGV display and is shown in additional file 13. In this case, although there is a *KRAS *DNA point mutation indicating a non-synonymous G13D, it is not reflected into the transcriptome and therefore, will not be translated to protein. Since a mutated *KRAS *protein is the drug target, a planned therapeutic assignment, which is many times based solely on DNA gene mutational data, may now be reconsidered given the integrated transcriptome findings.

**Figure 7:**
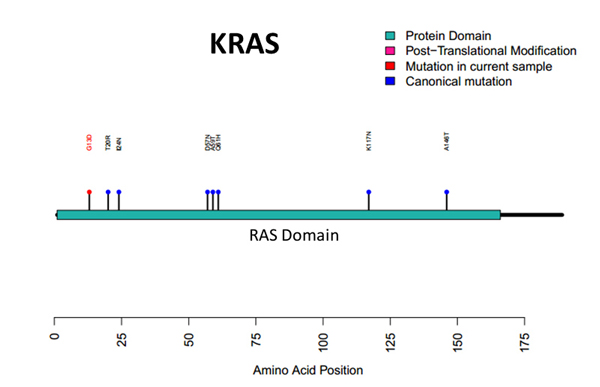
***KRAS *Protein Plot**. Automation is utilized to generate a "lolliplot", which illustrates the discovered amino acid changes for *KRAS *in the context of previously reported protein mutations and known domains. The G13D hotspot mutation found in the experiment is colored red. Other canonical mutations are colored blue. The RAS protein domain is colored green. Automation was achieved by a custom interface, along with R, the shiny package and Plot Protein.

Additional file 14 shows IGV validation of a novel splice isoform (CUFF.19733.1). Although it is from the normal pool it is shown for illustrative purposes. The novel exon is circled in purple, and the isoform with closest similarity is ENST00000311936. Figure 7 shows automation utilizing R with the shiny package and Plot Protein [25] for the *KRAS *G13D hotspot mutation. A "lolliplot" is produced that demonstrates amino acid changes in the context of previously reported mutations and protein domains. The G13D mutation found in our experiment is colored red. Other canonical mutations are colored blue. The RAS protein domain is colored green.

*TP53 *is a key tumor suppressor gene and known as the guardian of the genome. Cells that have a functional p53 pathway are less likely to transform. Genomic instability is a cardinal finding in p53 mutant cells. When *TP53 *is deleted in MM, a patient's likely survival becomes significantly shortened [26]. Classical tumor suppressor genes are usually characterized by nonsense or frame shift mutations that lead to truncated non-functional proteins. However, over 75% of all p53 mutations are missense and result in a single amino acid substitution. Accumulation of p53 mutants, to the point of showing a gain of function, can be seen because the mutant molecules can be more stable than the wild-type, and thus accrue [27-29].

Figure 3 shows the Cuffdiff Chart (**subfigure A**) and table (**subfigure B**) entries for *TP53*. Both the automatically generated bar chart and *Value 2 *(tumor) table entry clearly show *TP53 *is significantly overexpressed vs. normal. Additional file 15 shows a triple integration of experimental data sets from WES, Cuffdiff isoforms, and microarray. The red box is used to illustrate the seven *TP53 *transcript isoforms, which are associated with the appropriate non synonymous mutation (C277F or C266F or C145F) depending on how the exon with the point mutation (G > T) was spliced.

Approximately 90% of missense mutations for *TP53 *occur in the DNA binding domain, which spans amino acids 102-292, and all candidate SNVs in this experiment also fall in this region. The tumor depth is 82 reads and allelic frequency is 95%. By a single click on any of the TP53 entries, we can jump directly into IGV, and begin to validate and further explore this SNV. This is illustrated in the figure found in additional file 16. Since TP53 is coded on the reverse strand the point mutation appears as an "A" rather than "T", in IGV under the WES aligned reads section. In this case the RNA-seq aligned reads show concordance with the DNA findings. Together they provide stronger evidence for this biological finding. This important experimental data discovery was validated quickly due to the integrative and exploratory abilities of our system.

When *TP53 *is deleted, the effect and clinical ramifications are understood in MM and other cancers. However, the implications of *TP53 *missense mutations are not fully understood, and clearly may be important for therapeutic assignments and assessing prognosis. Thus, tools and approaches to track and study *TP53 *and other relevant cancer mutations are needed. Our system provides a small step in this direction.

## Conclusions

There is much to learn about the complexities of cancer genomes and NGS approaches are enabling a new understanding. As molecular profiling technologies evolve, it is critical to leverage what has been learned from historic techniques (e.g., microarrays) as new disruptive approaches (e.g., NGS) continue to gain a scientific presence and become a significant modality in clinical workflows (e.g., MySeqDx). These new and rapid changes are now reflected to the point that the science of computational biology is now driving many aspects of traditional "bench research". For instance, it is now common to use PCR to cross-validate specific computational findings. This is especially true in cancer. Furthermore, it is established that molecular heterogeneity results in complex genetic landscapes and are a common and confounding problem with many cancers. Demonstrated in this study are multi-modality integrative approaches, using *KRAS *and *TP53 *as examples, aimed to combat the heterogeneity dilemma, and bring more clarity to complex NGS studies.

The novel aspects of our approach are the abilities allowing for a ***rapid integration, exploration, discovery and validation ***of large and complex experimental data from a wide variety of NGS and microarray molecular profiling modalities, and are independent of any particular cancer type. Supported comparisons include: **i**) RNA-seq vs. Microarray, **ii) **WES vs. Microarray, **iii) **WES vs. RNA-seq, **iv) **WES vs. RNA-seq vs. Microarray (triple integration). The triple integration approach was illustrated in this study because it is the most difficult. To our knowledge, there is no existing software suite available, commercial or open source, which provides the triple integration feature. These abilities enable a more informed view of the intricate details present in the complex cancer biology, as was demonstrated with the *KRAS *and *TP53 *findings. The integrative *KRAS *findings indicate that the hotspot G13D DNA mutation does not appear to be translated into protein, thus negating the need for an expensive medicine targeting this particular mutation. *TP53 *integrative findings clearly displayed concordance between the DNA and RNA-seq transcriptome results and thus strengthen the biological evidence of the missense mutation.

There is a need to capture and model salient cancer biology knowledge gained from historic approaches. We continue to lay the groundwork for such endeavours concerning MM and other cancer types. It is our hope that such efforts will advance our understanding of the underlying cancer biology of MM, improve patient outcomes, and begin to further approach the concept of a cure.

## Competing interests

The authors declare that they have no competing interests.

## Authors' contributions

DJJ and MAB conceived and designed the study. MAB, SSC, EAP, CJH and DJJ performed the experiments and analyses. DJJ, MAB, SSC and EAP designed the software. MAB, SSC, and EAP implemented the software. DJJ and MAB wrote the manuscript. All authors approved the manuscript.

## Supplementary Material

Additional file 1 Click here for file

Additional file 2 Click here for file

Additional file 3 Click here for file

Additional file 4 Click here for file

Additional file 5 Click here for file

Additional file 6 Click here for file

Additional file 7 Click here for file

Additional file 8 Click here for file

Additional file 9 Click here for file

Additional file 10 Click here for file

Additional file 11 Click here for file

Additional file 12 Click here for file

Additional file 13 Click here for file

Additional file 14 Click here for file

Additional file 15 Click here for file

Additional file 16 Click here for file

## Declarations

Publication of this work was support by Program Project Grant CA 55813, from the National Institutes of Health (NIH), National Cancer Institute (NCI), Bethesda, MD, USA.

This article has been published as part of *BMC Bioinformatics *Volume 15 Supplement 11, 2014: Proceedings of the 11th Annual MCBIOS Conference. The full contents of the supplement are available online at http://www.biomedcentral.com/bmcbioinformatics/supplements/15/S11.

## References

[B1] Johnsen JM Nickerson DA Reiner AP Massively parallel sequencing: the new frontier of hematologic genomics Blood 2013 122 19 3268 3275 10.1182/blood-2013-07-460287 24021669 PMC3953088

[B2] Collins FS Hamburg MA First FDA authorization for next-generation sequencer N Engl J Med 2013 369 25 2369 2371 10.1056/NEJMp1314561 24251383 PMC5101955

[B3] Kandoth C McLellan MD Vandin F Ye K Niu B Lu C Xie M Zhang Q McMichael JF Wyczalkowski MA Mutational landscape and significance across 12 major cancer types Nature 2013 502 7471 333 339 10.1038/nature12634 24132290 PMC3927368

[B4] Mirnezami R Nicholson J Darzi A Preparing for precision medicine N Engl J Med 2012 366 6 489 491 10.1056/NEJMp1114866 22256780

[B5] Chapman MA Lawrence MS Keats JJ Cibulskis K Sougnez C Schinzel AC Harview CL Brunet JP Ahmann GJ Adli M Initial genome sequencing and analysis of multiple myeloma Nature 2011 471 7339 467 472 10.1038/nature09837 21430775 PMC3560292

[B6] Walker BA Wardell CP Melchor L Hulkki S Potter NE Johnson DC Fenwick K Kozarewa I Gonzalez D Lord CJ Intraclonal heterogeneity and distinct molecular mechanisms characterize the development of t(4;14) and t(11;14) myeloma Blood 2012 120 5 1077 1086 10.1182/blood-2012-03-412981 22573403

[B7] Morgan GJ Walker BA Davies FE The genetic architecture of multiple myeloma Nature reviews Cancer 2012 12 5 335 348 10.1038/nrc3257 22495321

[B8] Lohr JG Stojanov P Carter SL Cruz-Gordillo P Lawrence MS Auclair D Sougnez C Knoechel B Gould J Saksena G Widespread genetic heterogeneity in multiple myeloma: implications for targeted therapy Cancer cell 2014 25 1 91 101 10.1016/j.ccr.2013.12.015 24434212 PMC4241387

[B9] Wang Z Gerstein M Snyder M RNA-Seq: a revolutionary tool for transcriptomics Nature reviews Genetics 2009 10 1 57 63 10.1038/nrg2484 19015660 PMC2949280

[B10] Chavan SS Bauer MA Peterson EA Heuck CJ Johann DJ Jr Towards the integration, annotation and association of historical microarray experiments with RNA-seq BMC bioinformatics 2013 14 Suppl 14 S4 10.1186/1471-2105-14-S14-S4 24268045 PMC3851429

[B11] Shaughnessy JD Zhan F Burington BE Huang Y Colla S Hanamura I Stewart JP Kordsmeier B Randolph C Williams DR A validated gene expression model of high-risk multiple myeloma is defined by deregulated expression of genes mapping to chromosome 1 Blood 2007 109 6 2276 2284 10.1182/blood-2006-07-038430 17105813

[B12] Shaughnessy JD Qu P Usmani S Heuck CJ Zhang Q Zhou Y Tian E Hanamura I van Rhee F Anaissie E Pharmacogenomics of bortezomib test-dosing identifies hyperexpression of proteasome genes, especially PSMD4, as novel high-risk feature in myeloma treated with Total Therapy 3 Blood 2011 118 13 3512 3524 10.1182/blood-2010-12-328252 21628408 PMC3186329

[B13] Zhan F Huang Y Colla S Stewart JP Hanamura I Gupta S Epstein J Yaccoby S Sawyer J Burington B The molecular classification of multiple myeloma Blood 2006 108 6 2020 2028 10.1182/blood-2005-11-013458 16728703 PMC1895543

[B14] Zhan F Hardin J Kordsmeier B Bumm K Zheng M Tian E Sanderson R Yang Y Wilson C Zangari M Global gene expression profiling of multiple myeloma, monoclonal gammopathy of undetermined significance, and normal bone marrow plasma cells Blood 2002 99 5 1745 1757 10.1182/blood.V99.5.1745 11861292

[B15] Davis S Meltzer PS GEOquery: a bridge between the Gene Expression Omnibus (GEO) and BioConductor Bioinformatics 2007 23 14 1846 1847 10.1093/bioinformatics/btm254 17496320

[B16] Bolger AM Lohse M Usadel B Trimmomatic: a flexible trimmer for Illumina sequence data Bioinformatics 2014 24695404 10.1093/bioinformatics/btu170 PMC4103590

[B17] Li H Durbin R Fast and accurate short read alignment with Burrows-Wheeler transform Bioinformatics 2009 25 14 1754 1760 10.1093/bioinformatics/btp324 19451168 PMC2705234

[B18] Lunter G Goodson M Stampy: a statistical algorithm for sensitive and fast mapping of Illumina sequence reads Genome Res 2011 21 6 936 939 10.1101/gr.111120.110 20980556 PMC3106326

[B19] DePristo MA Banks E Poplin R Garimella KV Maguire JR Hartl C Philippakis AA del Angel G Rivas MA Hanna M A framework for variation discovery and genotyping using next-generation DNA sequencing data Nat Genet 2011 43 5 491 498 10.1038/ng.806 21478889 PMC3083463

[B20] Saunders CT Wong WS Swamy S Becq J Murray LJ Cheetham RK Strelka: accurate somatic small-variant calling from sequenced tumor-normal sample pairs Bioinformatics 2012 28 14 1811 1817 10.1093/bioinformatics/bts271 22581179

[B21] Cingolani P Platts A Wang le L Coon M Nguyen T Wang L Land SJ Lu X Ruden DM A program for annotating and predicting the effects of single nucleotide polymorphisms, SnpEff: SNPs in the genome of Drosophila melanogaster strain w1118; iso-2; iso-3 Fly (Austin) 2012 6 2 80 92 10.4161/fly.19695 22728672 PMC3679285

[B22] Thorvaldsdottir H Robinson JT Mesirov JP Integrative Genomics Viewer (IGV): high-performance genomics data visualization and exploration Briefings in bioinformatics 2013 14 2 178 192 10.1093/bib/bbs017 22517427 PMC3603213

[B23] Lee HC Shah JJ Orlowski RZ Novel approaches to treatment of double-refractory multiple myeloma American Society of Clinical Oncology educational book / ASCO American Society of Clinical Oncology Meeting 2013 23714530 10.1200/EdBook_AM.2013.33.e302 PMC3762449

[B24] Griffith M Griffith OL Coffman AC Weible JV McMichael JF Spies NC Koval J Das I Callaway MB Eldred JM DGIdb: mining the druggable genome Nature methods 2013 10 12 1209 1210 10.1038/nmeth.2689 24122041 PMC3851581

[B25] Turner T Plot protein: visualization of mutations Journal of clinical bioinformatics 2013 3 1 14 10.1186/2043-9113-3-14 23876180 PMC3724591

[B26] Drach J Ackermann J Fritz E Kromer E Schuster R Gisslinger H DeSantis M Zojer N Fiegl M Roka S Presence of a p53 gene deletion in patients with multiple myeloma predicts for short survival after conventional-dose chemotherapy Blood 1998 92 3 802 809 9680348

[B27] Goh AM Coffill CR Lane DP The role of mutant p53 in human cancer J Pathol 2011 223 2 116 126 10.1002/path.2784 21125670

[B28] Goldstein I Marcel V Olivier M Oren M Rotter V Hainaut P Understanding wild-type and mutant p53 activities in human cancer: new landmarks on the way to targeted therapies Cancer Gene Ther 2011 18 1 2 11 10.1038/cgt.2010.63 20966976

[B29] Brown CJ Lain S Verma CS Fersht AR Lane DP Awakening guardian angels: drugging the p53 pathway Nature reviews Cancer 2009 9 12 862 873 10.1038/nrc2763 19935675

