# Supplementary figures and images for: Leveraging the new with the old: providing a framework for the integration of historic microarray studies with next generation sequencing
Source: BMC Bioinformatics. 2014 Oct 21;15(Suppl 11):S3. doi: 10.1186/1471-2105-15-S11-S3 (PMC4251047; doi:10.1186/1471-2105-15-S11-S3)

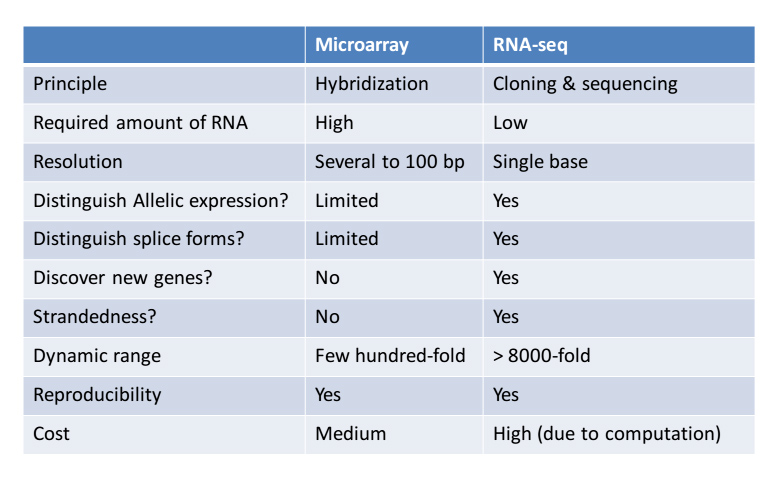

Supplement: Additional file 1 [file 1471-2105-15-S11-S3-S1.jpg]

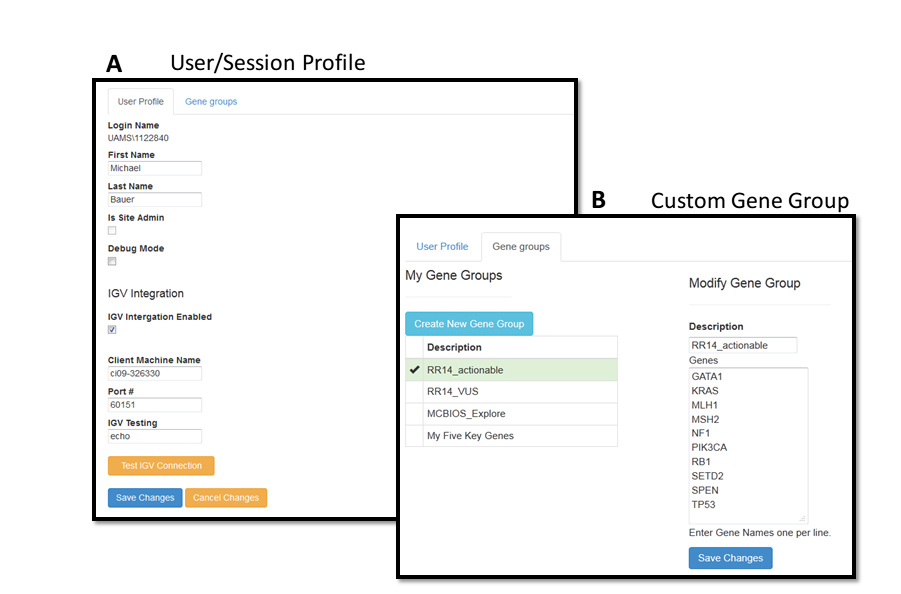

Supplement: Additional file 2 [file 1471-2105-15-S11-S3-S2.jpg]

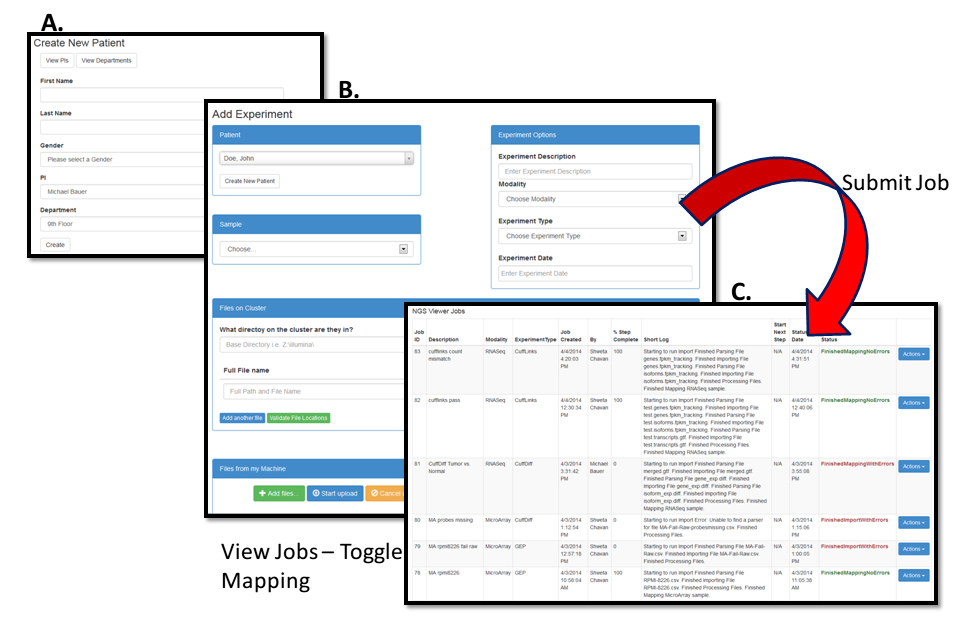

Supplement: Additional file 3 [file 1471-2105-15-S11-S3-S3.jpg]

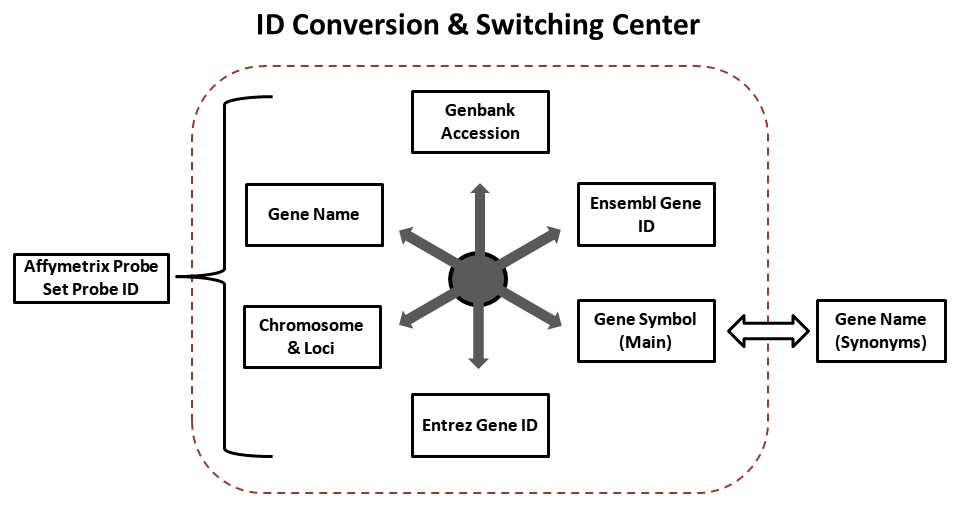

Supplement: Additional file 4 [file 1471-2105-15-S11-S3-S4.jpg]

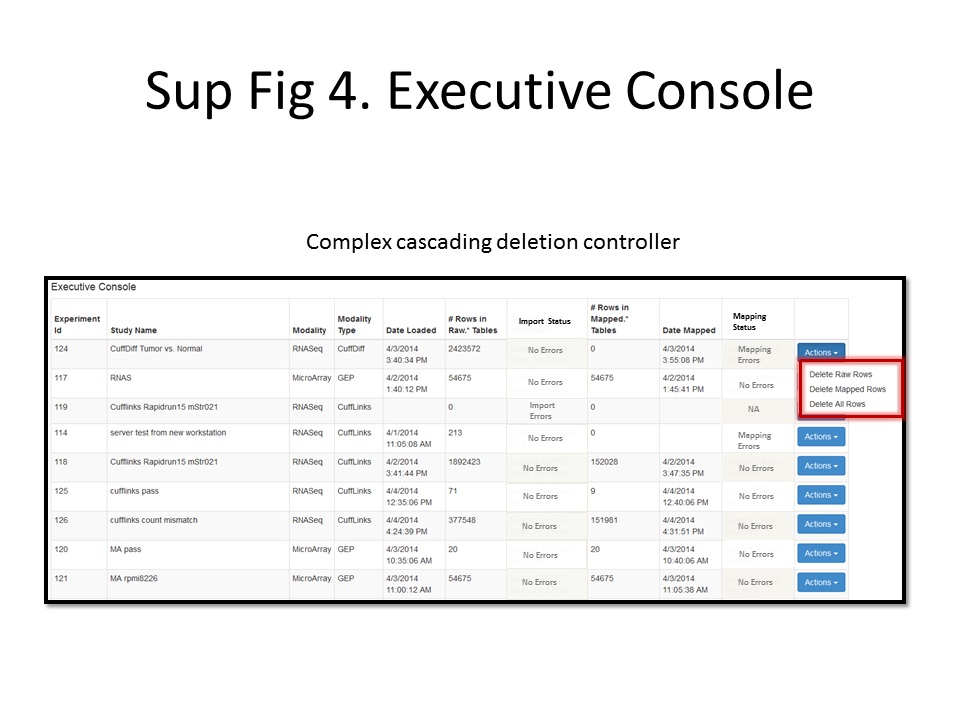

Supplement: Additional file 5 [file 1471-2105-15-S11-S3-S5.jpg]

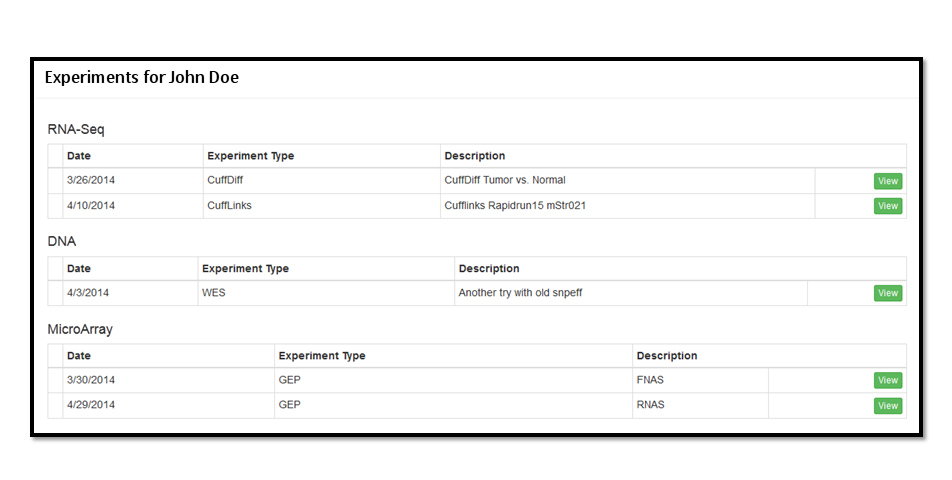

Supplement: Additional file 6 [file 1471-2105-15-S11-S3-S6.jpg]

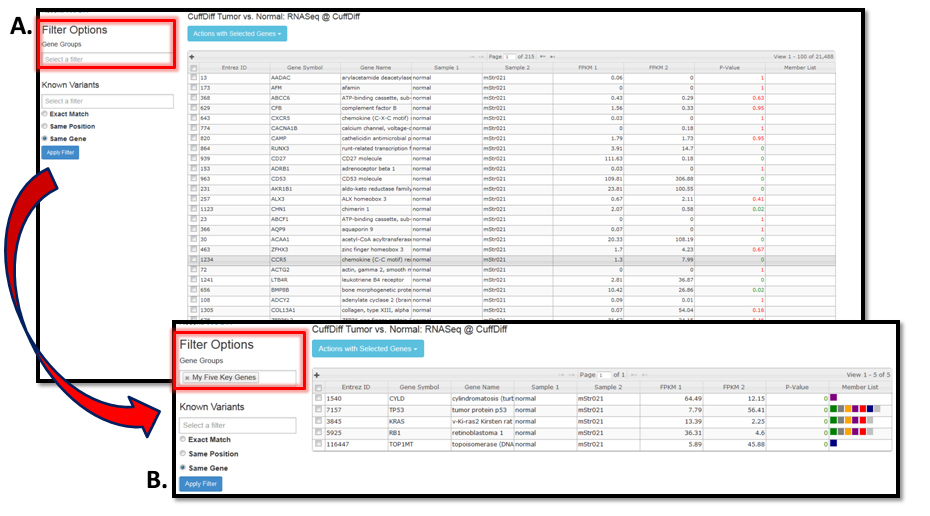

Supplement: Additional file 7 [file 1471-2105-15-S11-S3-S7.jpg]

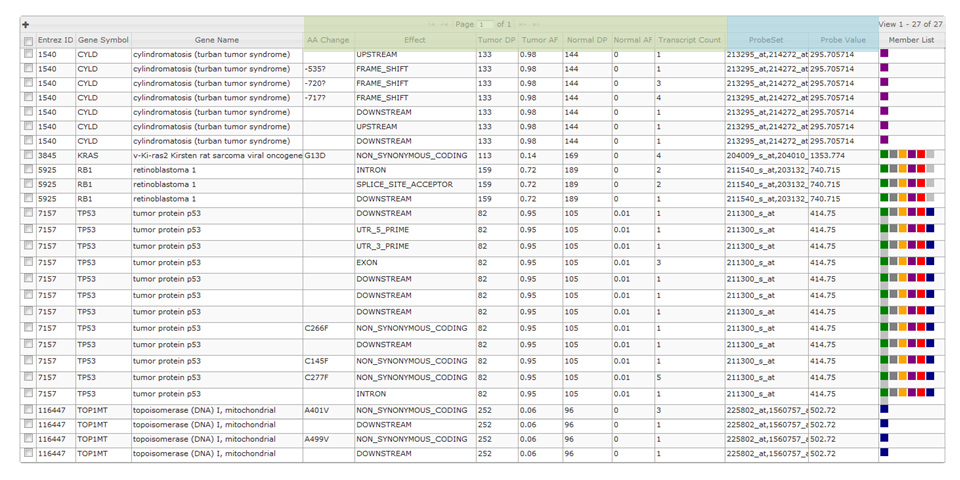

Supplement: Additional file 8 [file 1471-2105-15-S11-S3-S8.jpg]

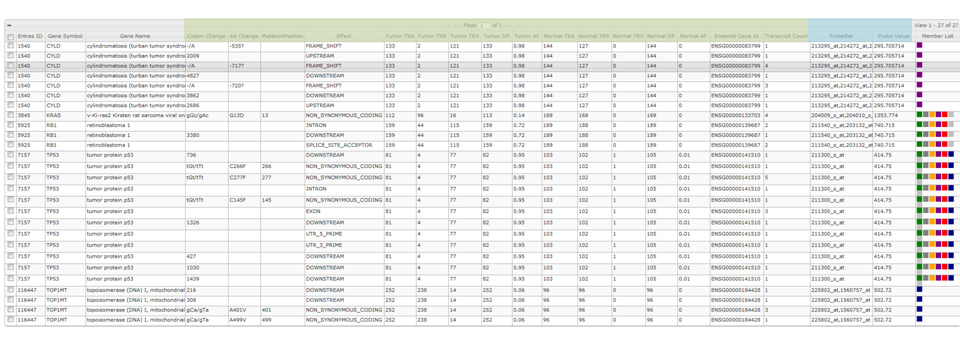

Supplement: Additional file 9 [file 1471-2105-15-S11-S3-S9.jpg]

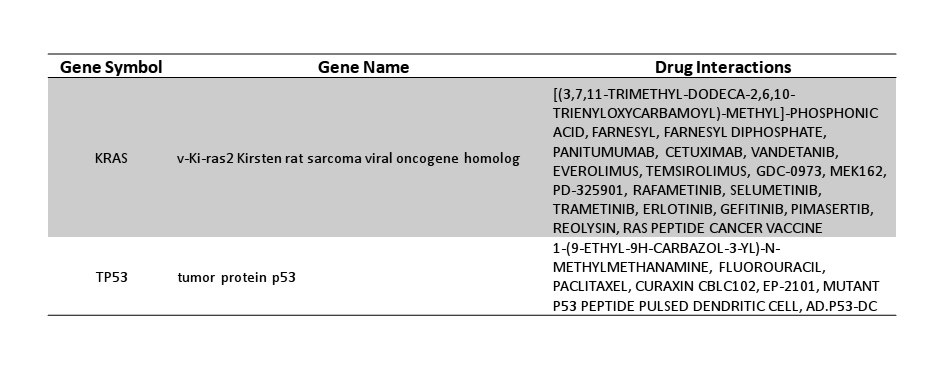

Supplement: Additional file 10 [file 1471-2105-15-S11-S3-S10.jpg]

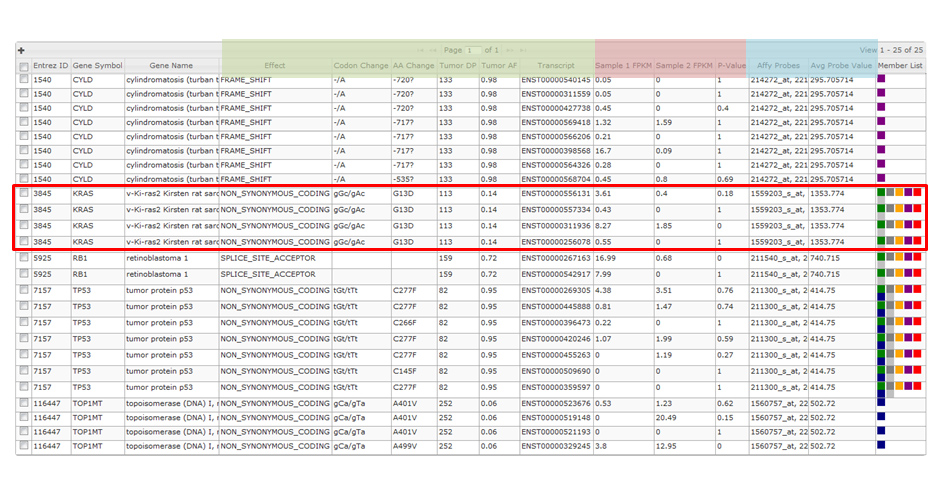

Supplement: Additional file 11 [file 1471-2105-15-S11-S3-S11.jpg]

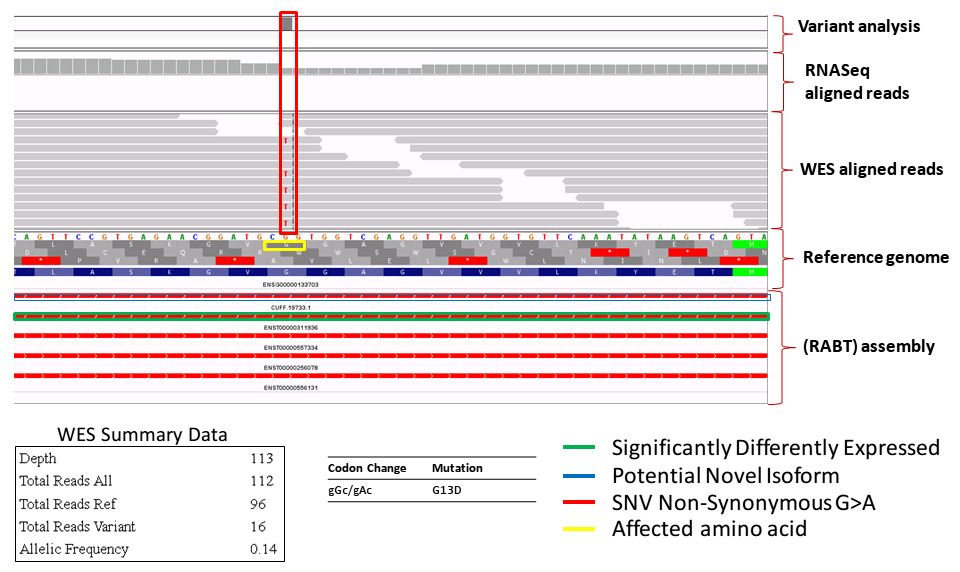

Supplement: Additional file 12 [file 1471-2105-15-S11-S3-S12.jpg]

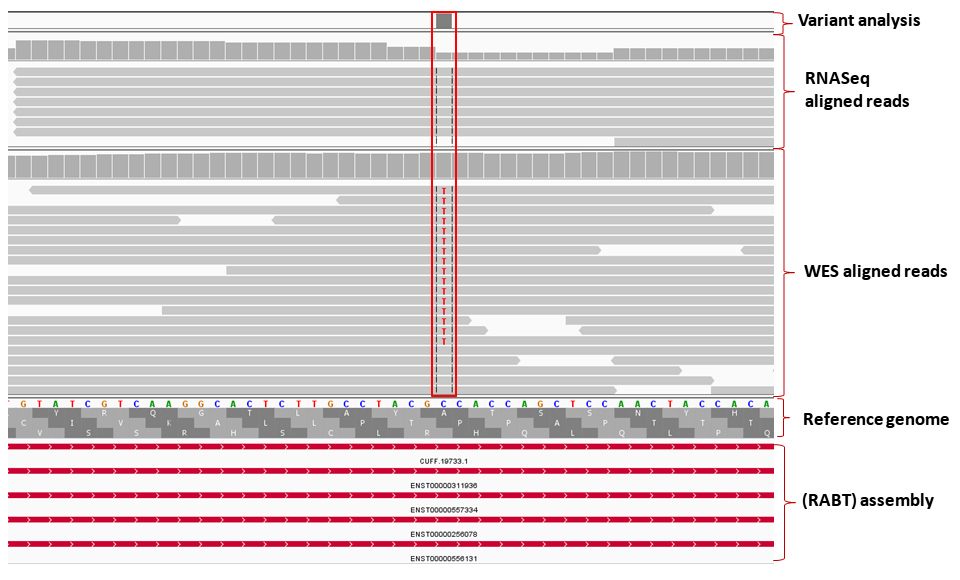

Supplement: Additional file 13 [file 1471-2105-15-S11-S3-S13.jpg]

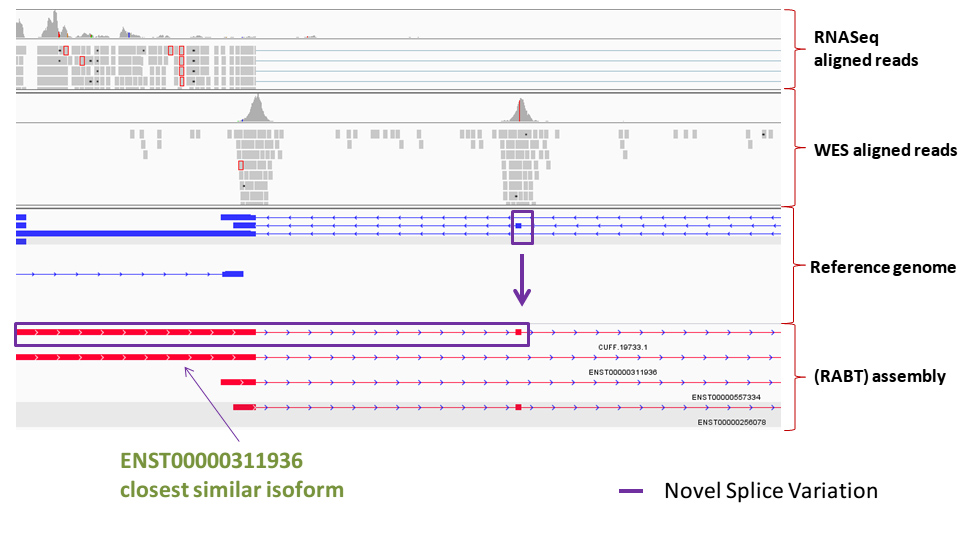

Supplement: Additional file 14 [file 1471-2105-15-S11-S3-S14.jpg]

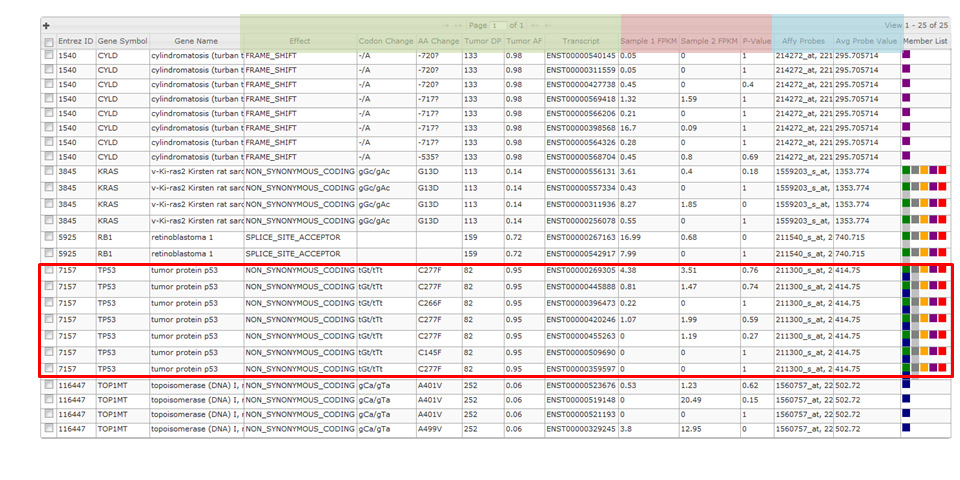

Supplement: Additional file 15 [file 1471-2105-15-S11-S3-S15.jpg]

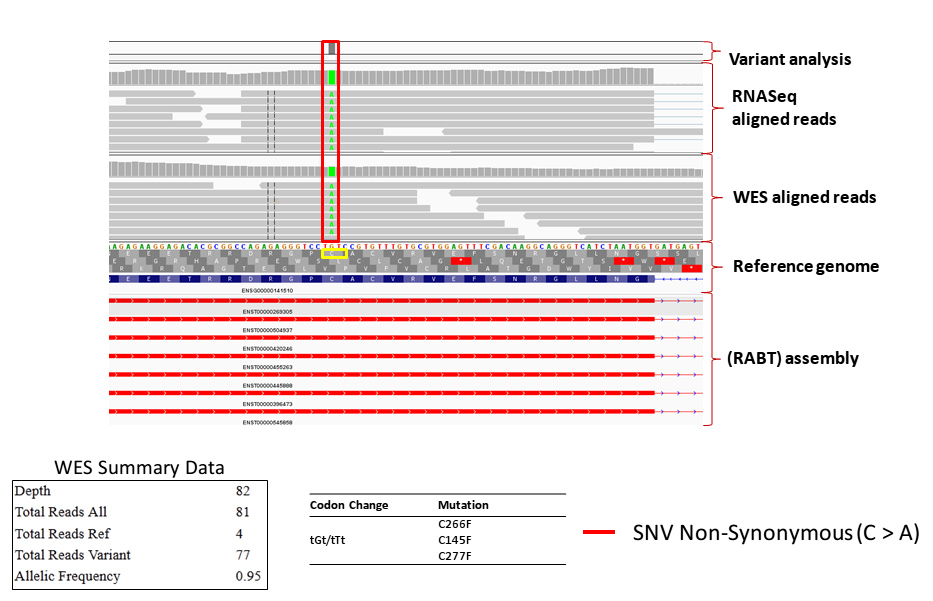

Supplement: Additional file 16 [file 1471-2105-15-S11-S3-S16.jpg]
